# Difference in Developmental Kinetics of Y-Specific Monoclonal Antibody Sorted Male and Female In Vitro Produced Bovine Embryos

**DOI:** 10.3390/ijms21010244

**Published:** 2019-12-30

**Authors:** Tabinda Sidrat, Rami Kong, Abdul Aziz Khan, Muhammad Idrees, Lianguang Xu, Marwa El Sheikh, Myeong-Don Joo, Kyeong-Lim Lee, Il-Keun Kong

**Affiliations:** 1 Department of Animal Science, Division of Applied Life Science (BK21 Plus), Gyeongsang National University, Jinju 52828, Gyeongnam, Korea; tabindasidrat06@gmail.com (T.S.); idrees1600@gmail.com (M.I.); xulianguang428@gmail.com (L.X.); marwa.el-sheikh@hotmail.com (M.E.S.); jmd1441@gmail.com (M.-D.J.); 0920-0728@hanmail.net (K.-L.L.); 2 Gyeongsang Animal Science Technology (GAST), Gyeongsang National University; Jinju-daero 501, Korea; mikbd1011@gmail.com; 3 Center for Discovery and Innovation, Hackensack University Medical Center, Nutley, NJ 07110, USA; azizkhanuop@gmail.com

**Keywords:** Bovine blastocyst, Y-specific monoclonal antibody, developmental kinetics, sex differences

## Abstract

Sex-related growth differences between male and female embryos remain an attractive subject for reproductive biologists. This study aimed to investigate the endogenous factors that play a crucial role in the pace of early development between male and female bovine embryos. Using sex pre-selected semen by Y-specific monoclonal antibodies for the production of bovine embryos, we characterized the critical endogenous factors that are responsible for creating the development differences, especially during the pre-implantation period between male and female embryos. Our results showed that at day seven, (57.8%) Y-sperm sorted in vitro cultured embryos reached the expanded blastocyst (BL) stage, whereas the X-sperm sorted group were only 25%. Y-BLs showed higher mRNA abundance of pluripotency and developmental competency regulators, such as *Oct4* and *IGF1-R*. Interestingly, Y-sperm sorted BLs had a homogeneous mitochondrial distribution pattern, higher mitochondrial membrane potential (**∆Ѱ_m_**), efficient OXPHOS (oxidative phosphorylation) system and well-encountered production of ROS (reactive oxygen species) level. Moreover, Y-blastocysts (BLs) showed less utilization of glucose metabolism relative to the X-BLs group. Importantly, both sexes showed differences in the timing of epigenetic events. All these factors directly or indirectly orchestrate the whole embryonic progression and may help in the faster and better quality yield of BL in the Y-sperm sorted group compared to the X counterpart group.

## 1. Introduction

Numerous studies have highlighted the differences in growth and developmental dynamics of the embryonic pre-implantation period between male and female mammalian embryos [1]. These observations show the differences in the pace of embryonic development after in vitro fertilization (IVF) treatment when cultured until the blastocyst (BL) stage, but the outcome has been inconclusive in several species [2,3]. Sperm sorting procedures and post-fertilization events affect the pre-implantation development, embryo quality, and outcomes of the gender ratio [3,4,5]. This subject is still a matter of debate.

The rapid growth of pre-implantation embryos is a manifestation of healthy embryos [6]. These rapidly developing embryos are preferentially selected for transfer, cryopreservation, and commercial breeding practices [5,7]. The faster growth rate of male relative to female embryos has led to concern about the in vitro culturing conditions and IVF treatment, which could result in a male-biased sex ratio [8]. The male embryos of different species, such as mice, pigs, cattle, and sheep, undergo a faster pre-implantation development relative to female embryos [1]. Different studies have reported that during human pre-implantation embryos from day two to the blastocyst stage, male embryos show a higher number of cells relative to female embryos [9]. This difference might arise due to the delay in the pre-implantation development of female embryos. The phenomenon of sex-specific embryo development kinetics has also been reported in human embryos [10,11]. So far, there is no clear answer about the developmental kinetics of male and female embryos.

The explicit answer to the growth difference between male and female embryos is not known. This difference might be an artifact of in vitro culturing conditions [2,3]. Genetic and epigenetic factors or the rate of metabolism and mitochondrial activity, which regulate the ATP (adenosine tri-phosphate) production, are the other factors that can generate a discrepancy between male and female embryos [12,13]. The in vitro culturing system is well established and has been used by many laboratories for decades, and has the ability to allow full in vitro development of oocyte harvested from an immature follicle [14]. However, in addition to the effect of in vitro culturing conditions on oocyte competence and its developmental rate, the interplay of spermatozoid genetic makeup affects the probability that an embryo shows a faster or slower developmental rate [3]. Environmental factors such as culture conditions influence the embryonic, fetal, and placental developmental in cattle, resulting in a higher incidence of male newborns [15]. Under adverse culture conditions, a high proportion of female embryos reach the morula stage but fail to advance to the blastocyst stage [16]. Females have two active X chromosomes up to the expanded BL stage. The extra X chromosome gene transcripts might be responsible for unbalanced metabolism and make them susceptible to developmental retardation [4,17].

However, regarding the differences in the growth of male and female in the pre-implantation period of bovine embryos, the current data are inconclusive and conflicting. In the present study, we aim to reveal sex-specific embryonic development kinetics in response to endogenous factors via a comparison between bovine male and female embryos. To conduct this study, we obtain the sex of pre-selected embryos by sperm sex-sorting via a Y-specific monoclonal antibody that constitutes a rationale strategy to provide a sufficient number of embryos without any damage to the sperm DNA content. Our study shows that in vitro produced male embryos have a faster rate of development as compared to female embryos due to differences in the context of developmental competence, mitochondrial activity, epigenetic reprograming, and X-chromosome dosage compensation mechanism.

## 2. Results

### 2.1. Effect of Gender Sex on Blastocyst Development Kinetics, Competency, and Cell Proliferation

**Figure 1:**
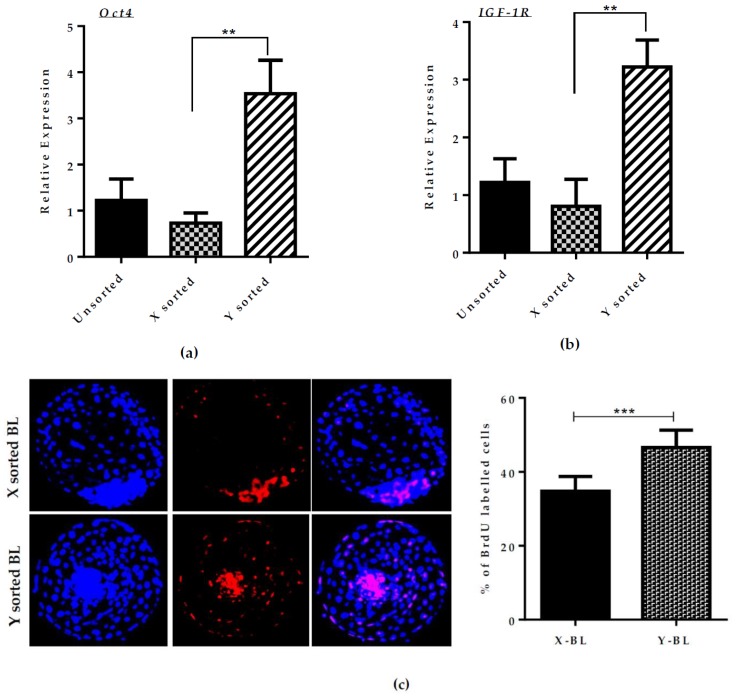
Differences in developmental competence and cell proliferation ratio during X- and Y-BL (blastocyst) development. Relative mRNA expression of (**a**) *OCT4* and (**b**) *IGF1-R* in unsorted, X-sorted, and Y sperm-sorted day seven BLs. (**c**) Immunofluorescence staining of 5-bromo-2′-deoxyuridine (BrdU) in X and Y sperm-derived day seven BL. Quantification of cell proliferation rate. Bar graph data represent means ± SEM (standard error of mean) from three independent sets of experiment, including *n* = 20 BLs per group in each replicate. ** p < 0.01; *** p < 0.001 indicates significant difference. Original magnification 100×.

Following post-fertilization, the timing of early BL appearance is usually associated with the speed of development in many species [6,13]. To analyze the difference in development kinetics among male and female sexed embryos, we compared the formation of an expanded BL ratio between two groups. Sex preselected embryo production via a Y-specific monoclonal antibody yielded more than 70% accuracy in male and female BL development [7]. Among the X- and Y-sorted embryo groups, more than 55% of the Y-sorted embryos rapidly developed to the expanded BL stage at day seven post-fertilization, whereas the X-sorted group produced only 25% expanded BLs at day seven (Table 1). To examine the effect of gender sex during embryo progression to the BL stage between the X- and Y-sorted embryos, we determined the relative transcript abundance of the developmental competency-related genes expression profile. The results of quantitative real-time PCR (polymerase chain reaction) analysis revealed that there is a significant difference in the expression level of many important genes, such as *Oct4* and *IGF1-R*, between BL derived from X- and Y-sorted spermatozoa groups (Figure 1A,B). To confirm the stability of *GAPDH* (glyceraldehyde 3-phosphate dehydrogenase) expression that was used as an internal control, its expression in both sexes was normalized with 18SrRNA. There was no big difference in the expression of *GAPDH* between the two sexes (Figure S1). The pluripotency marker gene *Oct4* was higher in the Y-sorted BLs group. The higher cell proliferation might be due to the higher cell number count. To verify this hypothesis, we performed a BrdU (5-bromo-2’-deoxyuridine) incorporation assay. Consistent with RT-PCR observation, the Y sperm sorted BLs group also had a higher cell proliferation ratio than X sperm-generated BLs (Figure 1C).

### 2.2. Effect of Gender Sex on Mitochondrial Functioning Status in Embryos

**Figure 2:**
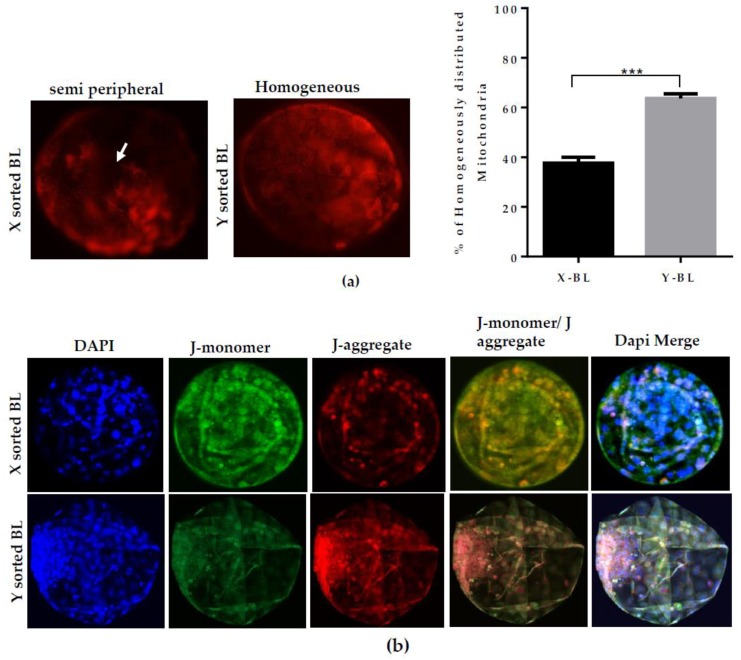

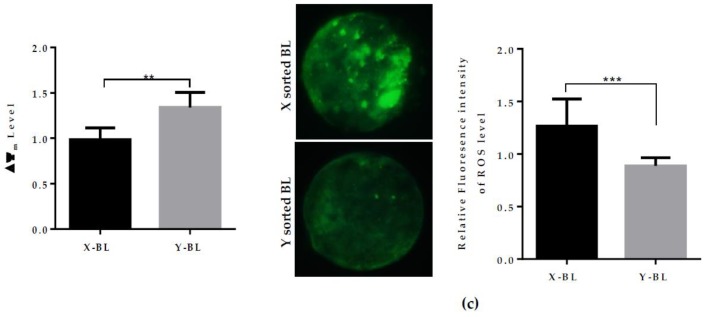
Difference in mitochondrial distribution, ∆Ѱ_m_, and ROS level during the development of X- and Y-sorted BLs. (**a**) Representative images of Mitotracker (Red) staining showing X-BL with semiperipheral and Y-BL with homogeneous distribution patterns of mitochondria. Data in graph represent the percentage of homogeneously distributed mitochondria in the X- and Y-BL groups. (**b**) Expression of J-monomer (green) and J-aggregates (red) was analyzed by JC-1 staining to measure the mitochondrial **∆Ѱ_m_** in X- and Y-BL using confocal microscopy. Quantification for relative fluorescence intensities in both X- and Y sperm-derived BLs are presented in graph. (**c**) DCHDFA (2’, 7’-dichlorodihydrofluorescein diacetate) staining for the generation of ROS level in X- and Y-BL. Quantification of fluorescence intensities is shown in graph. Bar graphs represent means ± SEM from three separate experiments with day seven BLs, *n* = 15 per group in individual sets of assays. ** p < 0.01; *** p < 0.001. Original Magnification 100×.

Mitochondrial membrane potential (**∆Ѱ_m_**) is attributed to mitochondrial metabolic activity for the generation of ATP. ATP is critical for oocyte maturation, fertilization, and subsequent embryonic development [18]. To examine the effect of gender sex during development, we analyzed the mitochondrial distribution pattern, mitochondrial ∆Ѱ_m_, and generation of reactive oxygen species (ROS) in X- and Y-sorted BLs. Male BLs showed a uniform distribution of mitochondria compared to female BLs. The female BLs had a semi-peripheral distribution (Figure 2A). In X-BLs, the ratio of J-aggregate versus J-monomer was lower compared to Y-sorted BLs (Figure 2B). Low mitochondrial activity cannot actively eliminate the ROS from the cells [13,18]. The accumulation of ROS level also arrests the development at early stages [19]. Importantly, we also detected high florescence signal for ROS activity in the X-BLs compared to the Y-BLs (Figure 2C). These findings suggest that mitochondrial metabolic activity is critical for BL growth and maturation. Furthermore, both mitochondrial metabolic activity and ROS levels are important factors for the developmental competency of bovine BLs.

### 2.3. Mitochondrial OXPHOS Deficiency Attributed to Slower Development Progression of X Sperm-Sorted BLs

**Figure 3:**
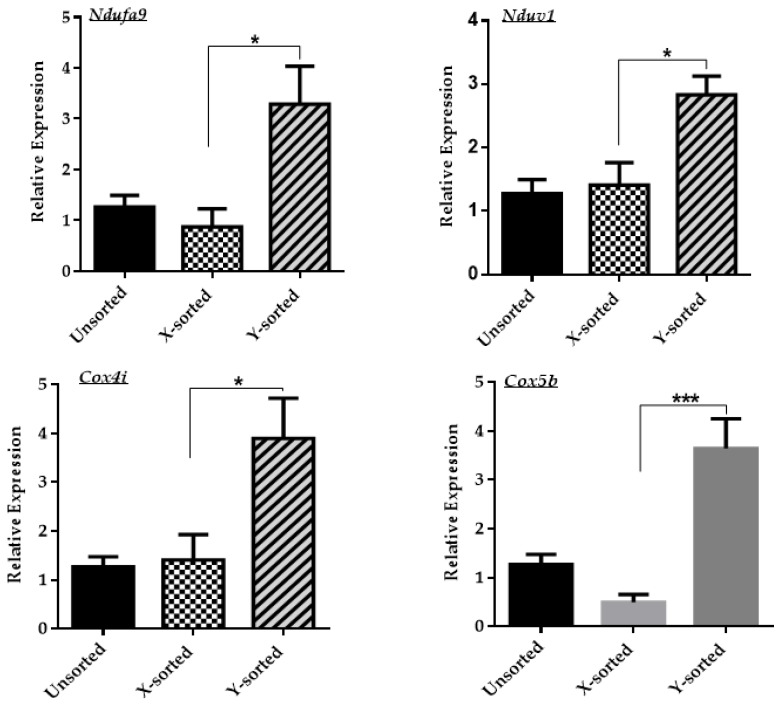
Differences in the mitochondrial OXPHOS system attributed to the development of X- and Y-BLs. Relative mRNA expression analysis of mitochondrial OXPHOS subunit genes, *Ndufa9, Ndufv1, Cox4i, Cox5b* in unsorted, X-, and Y-sperm-sorted BLs at day seven. Data in the bar graphs represent the means ± SEM from three independent sets of experiments. * p < 0.05; *** p < 0.001.

Based on our current analysis, the X chromosome-derived BL group had a lower **∆Ѱ_m_** and relatively higher ROS level than the Y sperm-derived BL group. Based on this observation, we speculated that X sperm-derived embryos might have a perturbed mitochondrial OXPHOS (oxidative phosphorylation) system compared to the Y group. A deficiency in the mitochondrial OXPHOS system results in the production of superoxide. Superoxide disturbs the inner mitochondrial **∆Ѱ_m_** and subsequently down regulates the expression of genes associated with ATP synthesis [20,21]. To determine the mitochondrial OXPHOS status, we analyzed the relative transcript level of four mammalian OXPHOS complex subunit genes *Ndufa9, Ndufv1, Cox5, Cox4i.* The expression of OXPHOS-related genes was significantly higher in Y sperm-produced BLs compared to the X sperm-generated BLs group (Figure 3). During the pre-implantation of embryonic development, there is a gradual shift in the process of metabolism. This shift increases the mitochondrial oxidative phosphorylation [21]. Based on previous reports and our current results, we suggest that a less efficient mitochondrial OXPHOS system is one of the possible reasons that affects the speed of development during embryonic progression in the X sperm-derived in vitro BLs.

### 2.4. Differences in Preimplantation Development are Attributed to Cell Metabolism and Apoptotic Index of Sex-Sorted BL

**Figure 4:**
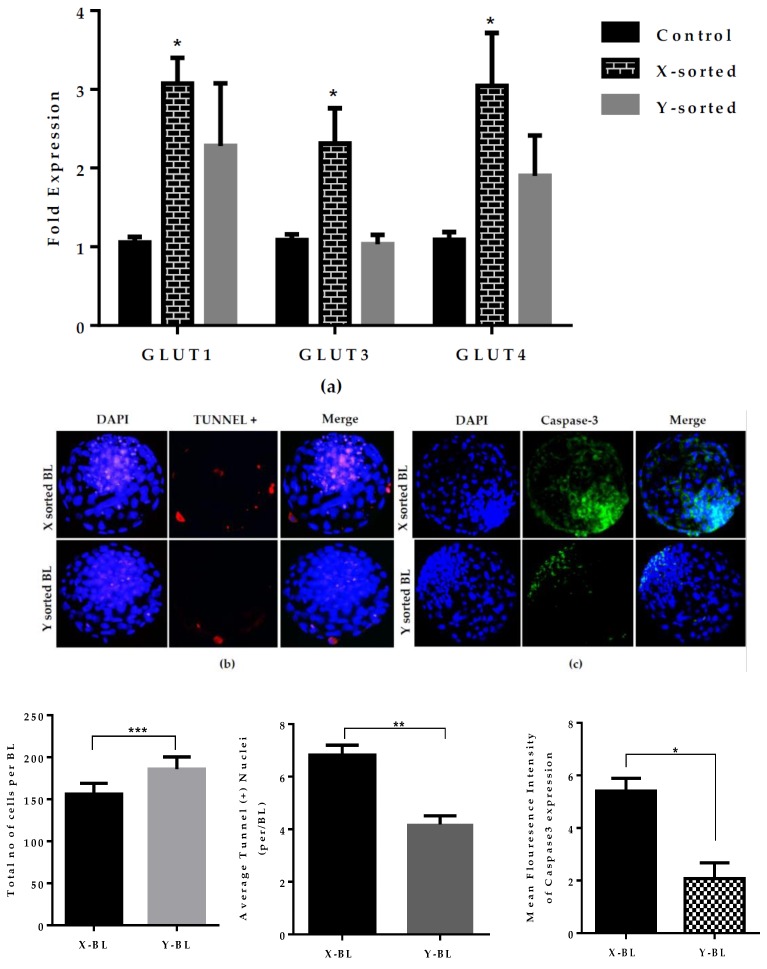
Differences in cell metabolism and apoptosis level on the quality and development kinetics of X- and Y-sorted BL. (**a**) Relative mRNA level of *GLUT1, GLUT3, GLUT4* in control, X-, and Y-sperm generated day seven BL groups. (**b**) Fluorescence microscope image of TUNEL-positive cells (red) and DAPI (4,6-diamidino-2-phenylindole) (blue) shown in X- and Y-BL at day seven. White arrows indicate the apoptotic cells in nuclei. (**c**) Representative fluorescent image showing Caspase-3 expression in X- and Y-BL. Graphical data represent the quantification of fluorescent images of X- and Y-BL groups. Data in the bar graphs represent the means ± SEM from three independent sets of experiments including *n* = 20 BLs per group in each replicate. * p < 0.05; ** p < 0.01; *** p < 0.001. Original magnification 100×.

Cell metabolism and apoptosis are important factors that are used as indicative tools for determining the embryonic development potential [14,22]. We assumed that cell metabolism and apoptosis level could be among the reasons for the tendency of male embryos to develop faster than female embryos. Previously, it was reported that higher glucose consumption also impacts growth-related sex differences [23]. To determine the growth differences between sexes, we analyzed the expression profile of metabolism-related genes *Glut1, Glut3, and Glut4*. These are known as glucose transport genes [24,25]. Higher expression of all *Glut* genes was found in X sperm-sorted BLs compared to the Y sperm-sorted BLs (Figure 4A). The expression of high mRNA of *Gluts* genes in X-BLs suggests that female embryos have a higher glucose consumption rate compared to the male embryos. Many studies have reported that cells in the higher glucose environment are more prone to cell death, which negatively affects the growth and development of the embryos [26]. We therefore analyzed the apoptotic ratio between the X and Y sperm-generated BLs (Figure 4B). The percentage of TUNEL-positive (terminal deoxynucleotidyl transferase (TdT) 2′-deoxyuridine, 5′-triphosphate (dUTP) nick-end labeling) nuclei per blastocyst was observed to be higher in the X-BLs group compared to the Y-BLs group. This was further substantiated by the immunofluorescence intensity of a pro-apoptotic gene, such as Caspase-3 expression (Figure 4C). The in vitro fertilized X sperm-derived BL group showed significantly higher Caspase-3 expression compared to the Y sperm-generated BL group. These results suggest that X chromosome-bearing embryos utilize higher glucose metabolism to support their growth and development. This results in a higher risk of apoptosis and ultimately slower development potential as compared to their male counterparts.

### 2.5. Aberrant Expression of X-Linked Genes Generate Sex-Related Differences in Embryonic Developmental Dynamics

**Figure 5:**
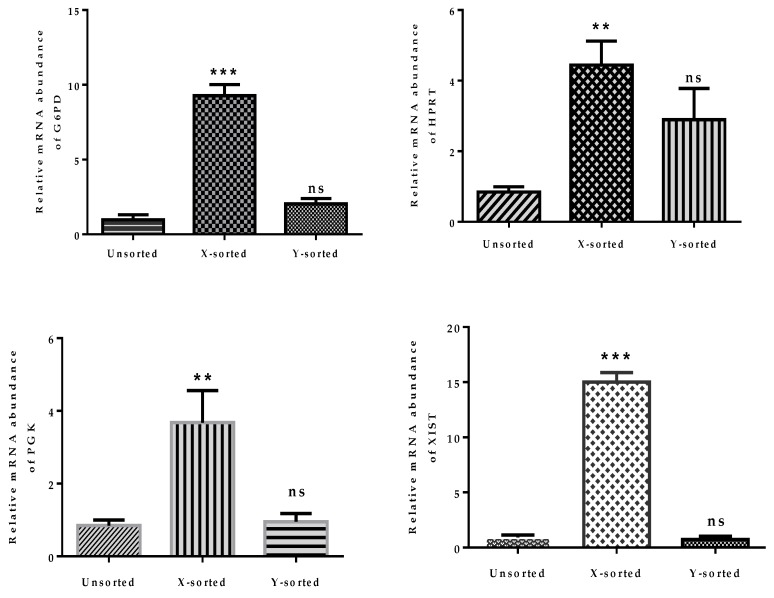
Relative mRNA expression of X-chromosome linked genes in X and Y sperm-sorted BLs. Quantitative real-time PCR analysis showing the mRNA expression level of X-chromosome linked genes, *G6PD, HPRT, PGK,* and *XIST* in an unsorted, X, and Y sperm-sorted BL. Data in the bar graphs represent the means ± SEM from three independent sets of experiments. ** p < 0.01; *** p < 0.001. ns indicated the non-significant difference as compared to unsorted group.

Differences in the regulation of X-linked genes between sexes during early mammalian development is one of the factors that creates variation in the developmental kinetics of male and female BLs [12,15,27,28]. Our results showed that mRNA expression of *G6PD, HPRT, PGK* and *XIST* were significantly higher in female than in male IVF BL (Figure 5). The high expression of *XIST* RNA in the female BLs compared to male BLs showed the readily distinguishable characteristics of the X-sperm sorted BLs. However, a low expression of *XIST* RNA was also detected in the unsorted group, as it contained a mixed population of X and Y sperm-derived BLs. The expression of X-linked genes in Y sperm-sorted embryos and the unsorted group showed non-significant differences in their Ct values, as the unsorted group had both X and Y embryos, and also highlighted that the expression of the X-linked gene was exclusively expressed with higher mRNA abundance in the X sperm-sorted group. This result suggests that higher expression of the X-linked genes in the female BLs results in an imbalance of the X-chromosome dosage compensation mechanism, which ultimately affects the developmental speed of female BLs.

### 2.6. Epigenetic Reprogramming during Pre-Implantation Development of X- and Y-Sorted Embryos

**Figure 6:**
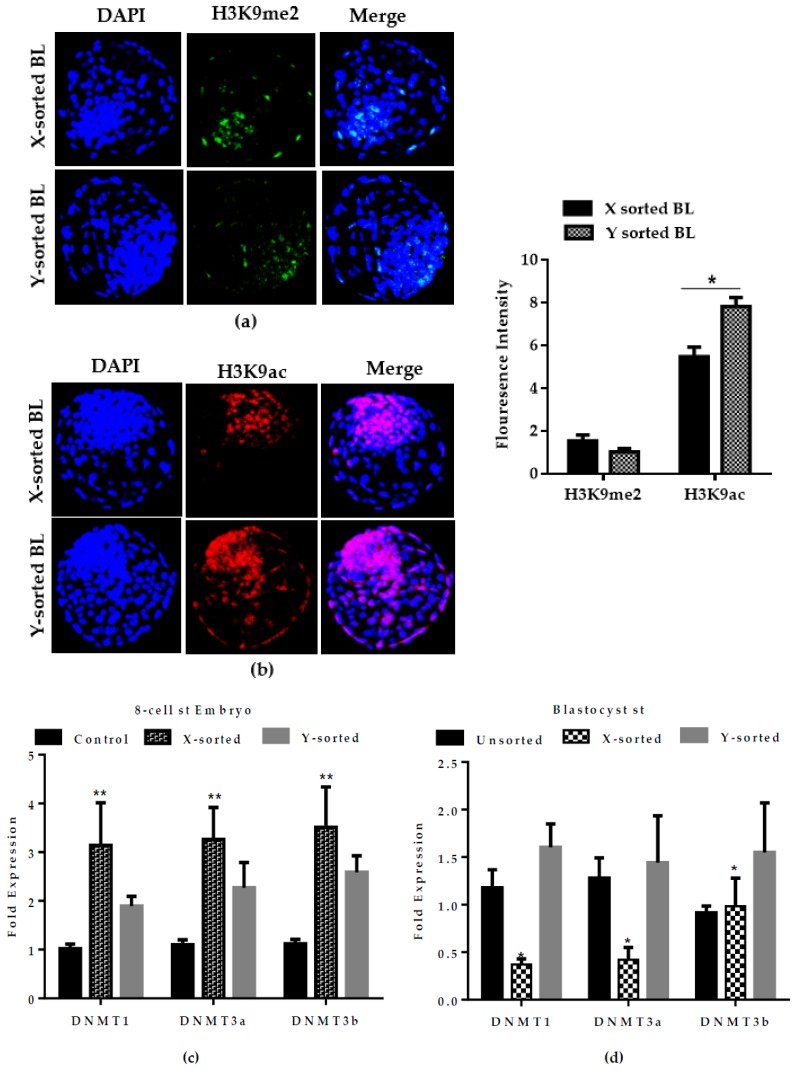
Epigenetic status during development of X and Y sperm-sorted BL. (**a**) Representative fluorescent images of H3K9me2 and (**b**) H3K9ac in X- and Y-BL. Mean fluorescence intensity of H3K9me2 and H3K9ac signals in the X- and Y-BL groups are presented in the graph. The experiment was performed in triplicate sets of experiment with 20 BLs per group in each replicate (**c,d).** Analysis of relative mRNA expression of *DNMTs* in X and Y sperm-sorted 8-cell stage embryo and day eight BLs. Data in the bar graphs represent the means ± SEM from three independent sets of experiments. * p < 0.05; ** p < 0.01. Original magnification 100×.

Genetic and epigenetic reprograming strongly influence the period of pre-implantation development [4,12]. The degrees of methylation and acetylation play imperative roles in establishing the pattern of gene transcription during the course of bovine embryonic development [4,29]. We hypothesized that epigenetic events might be a developmentally relevant factor that modulate the speed of embryonic progression, development competency, mitochondrial activity, and metabolism between X- and Y-chromosome-derived BL groups. We analyzed global H3K9me2 (transcriptional repression mark) and H3K9ac (transcriptional activation mark) in X and Y sperm-produced in vitro BLs. The immunofluorescence intensity of H3K9me2 showed no difference in the X and Y sperm-sorted BL groups, while the signal intensity of H3K9ac was found to be significantly higher in the Y sperm-sorted BL group (Figure 6A,B). The high expression of H3K9ac likely enhances the expression of vital genes that regulate the dynamics of development. Interestingly, the acetylation signal was higher in Y-BL than X-BL. The high acetylation level in Y-BL might enhance the expression of vital genes, which ultimately accelerate the speed of development compared to X-BLs. Moreover, the relative mRNA abundance of DNA methyltransferases *DNMT1, DNMT3a, DNMT3b* was found to be significantly higher in the X-embryo group compared to the Y sperm-derived 8-cell stage embryos. However, a lower expression level in the X group was observed as compared to the Y group at the BL stage (Figure 6C,D). High and low methylation and acetylation levels are important epigenetic alterations during early embryonic development and could be important factors causing differences in the development kinetics between male and female BLs.

## 3. Discussion

In the present study, we investigated the difference in developmental kinetics between X and Y sperm-sorted bovine embryos during the pre-implantation period. Previously, it was reported in several mammalian species, including bovine, that male embryos develop faster to the BL stage than the female embryos, by using crude methods of sperm sexing ([3,6]. This method can damage the DNA content of the sperm and modulate the outcome of the experiment. To resolve the conflicting results regarding the issue of differences in developmental kinetics of male and female in vitro cultured embryos, we preselected the sex of bovine embryos by using the WholeMom antibody. Sex sorting of embryos via a Y-specific monoclonal antibody yields 72% male BLs. This specificity was confirmed by Y-specific gene expression. Importantly, WholeMom-treated X sperm-sorted group through artificial insemination produced 76% female and 24% male in the field study [7]. This antibody exclusively binds to the head of Y-bearing spermatozoa and cause head-to-head agglutination of the Y sperms. It separates the bull semen into Y and X sperm milieu without causing any harm to the genetics of Y-chromosome-bearing spermatozoa. However, in our results, the cleavage rate was higher in the X-sorted group compared to the Y-sorted group (Table S1). The low cleavage rate in the Y-sorted sperm group was due to the WholeMom antibody, which causes agglutination of the Y spermatozoa and decreases the fertilization capacity by inhibiting the movement of Y sperm [7]. We found that BL expansion and hatching rate was consistently higher in the case of Y sperm-sorted embryos compared to X sperm-produced embryos. Based on this observation, we investigated the relative mRNA expression of development regulated genes *Oct4* and *Igf1-R.* The expression of these genes was significantly higher in the Y sperm-sorted group compared to the X sperm-sorted group (Figure 1A,B). This observation is consistent with a previous report [23]. *Oct4* is a major pluripotency regulator and is highly expressed in male embryos, which may explain the higher tendency of cell proliferation ratio in male embryos and consequently result in a faster and better quality of BL development. Overall, our results regarding the expanded BL yield at day seven suggested that Y sperm-produced in vitro embryos have a better quality and faster development compared to the X sperm-generated in vitro embryos.

During the preimplantation period, efficient activity of mitochondria is a determining factor in the speed of development between the sexes [19]. High mitochondrial activity or **∆Ѱ_m_** controls the level of ROS production. With higher **∆Ѱ_m_**, the conversion of free radical species O_2_ into less toxic oxidants such as H_2_O_2_ is more efficient_._ This maintains the low level of ROS in mitochondria. A higher level of ROS also interferes with the production of ATP, which is required to support the cell proliferation and differentiation event during blastulation [18]. Our results demonstrated that Y-BLs displayed a significantly higher **∆Ѱ_m_** and lower ROS level in contrast to the X-BLs (Figure 2B,C). The less efficient mitochondrial system might be the cause of decreased embryonic development competence and delay the speed of embryonic progression in female BLs. Many studies have reported the changes in the inner mitochondrial **∆Ѱ_m_** level and metabolite homeostasis, such as the generation of ROS, which is directly related to deficiencies in the mitochondrial OXPHOS system [30]. Deficiency in the mitochondrial OXPHOS system has a pronounced effect on the overall transcriptional response of several downstream genes that are modulated by the level of superoxide metabolites [20]. In our observations, altered mitochondrial membrane potential and a relatively increased production of ROS level in the X embryo group showed that male and female developing embryos have differential expression of mitochondrial OXPHOS genes. As shown in Figure 3, the Y sperm-sorted BL group displayed a higher expression of mitochondrial OXPHOS subunit genes compared to the X sperm-derived BLs. These results suggest that an inefficient mitochondrial OXPHOS system in the in vitro developed female embryo is one of the critical factors that might contribute to delayed embryonic development relative to male embryos. The failure of uncoupling and subsequent liberation of ROS level consequently disrupt the inner mitochondrial **∆Ѱ_m_** and cellular energy metabolism.

Mammalian embryos utilize glucose as a primary source of nutrients. Glucose is metabolized either oxidatively or through aerobic glycolysis to facilitate successive stages of development [31]. To determine the difference in the glucose utilization between the X and Y sperm-sorted BL groups, we quantitatively analyzed the expression of the *GLUTs* genes. *GLUTs* genes expression was significantly higher in the X sperm-derived BL group than in the Y sperm-derived BL group (Figure 4A). This result was in agreement with previous investigations that female embryo have higher glucose consumption than male embryos. Higher utilization of glucose ultimately stimulates the glycolysis, which disrupts the oxidative phosphorylation by altering the mitochondrial membrane potential and subsequently leads to the activation of apoptosis by the overproduction of ROS [22]. Our results also revealed that X-BLs have a significantly higher cell apoptotic ratio and caspase-3 expression than Y-BL group (Figure 4B,C). These observations are similar to previous reports on mouse embryos, showing that alterations in glucose metabolism lead to cell death [26]. Similarly, the high expression of *GLUTs* genes in the X-BL group reflects the disruption of metabolic normality, which could be associated with the arrest of development due to an unbalanced redox status.

The above observations, showing disturbed mitochondrial function and imbalanced glucose metabolism during pre-implantation stages of female embryonic development, strongly emphasized that dosage compensation of X-chromosome linked genes was not properly achieved. The relative mRNA abundance of four X-linked genes was significantly higher in X-BLs than in Y-BLs (Figure 5). Among these genes, two are important regulators of glycolysis and are involved in controlling the production of oxygen radicals (glucose 6-phosphate dehydrogenase (*G6PD*) and hypoxanthine phosphoribosyl-transferase (*HPRT*)) [23]. The differential expression of these genes was consistent with previous investigations in several species, including bovine, human, mouse, and porcine embryos, which showed that many X-linked genes at the BL stage were preferentially expressed in females rather than male BLs [4,15,28,32]. The X-chromosome dosage compensation mechanism by random inactivation of one X-chromosome is an essential process during the early phases of embryonic development. Failure to achieve complete inactivation during in vitro production would initiate epigenetic differences between female and male bovine BLs [33]. Moreover, a higher abundance of *DNMTs* transcript was detected in the early embryonic stage of X-embryos, while expression was decreased at the BL stage relative to the Y counterpart group (Figure 6C,D). DNA methylation is characterized as transcriptional repressive mark, and the higher mRNA abundance of *DNMTs* suggests that X-embryos possess a transcriptional repressive mark at an early stage, which arrests the development. The low expression of *DNMTs* at the BL stage suggests the presence of hypomethylation status in the X sperm-derived BL group. This observation suggest that X-chromosome inactivation was not fully accomplished during in vitro development. This result was consistent with previous reports [12,29]. Collectively, these observations highlighted that differences in the speed of development between X and Y sperm-derived BLs was modulated by epigenetic events during the preimplantation period. The result also suggests a possible reason for the presence of physiological and gene transcriptional differences between male and female developing embryos.

We determined several possibilities that accounted for a plausible explanation of how the differences in developmental kinetics between the sexes are established. Finally, we checked the probability of in vitro produced male embryos among the rapidly expanded BLs at day seven in an unsorted group. Day seven-collected samples showed a higher percentage of male developing BLs which expanded and hatched one day earlier than day eight BLs, which showed a higher percentage of females (Figure S2, Table S2). Collectively, our results showed that male embryos have a faster rate of development compared to the female embryos. Both sexes showed different mitochondrial functioning status, metabolism, and epigenetic events, which influence their development rate.

## 4. Materials and Methods

### 4.1. Oocyte Aspiration and In vitro Maturation

Ovaries from Korean native Hanwoo cows (Jinju-city, South Korea) were obtained from the local slaughterhouse under the legislation of Institutional Animal Care and Use Committee of Gyeongsang National University (Approval ID: GAR-110502-X0017; date: 02-05-2011), and placed in physiological saline (0.9% NaCl) at 37.5 °C. The ovaries were washed with fresh Dulbecco’s phosphate buffered saline (D-PBS) and COCs (cumulus oocyte complexes) were retrieved from follicles (2–8 mm diameter) by using an 18-gauge needle attached to a vacuum pump. Aspirated follicular fluid was expelled into dishes containing TL-HEPES medium (6.662 g/L sodium chloride, 0.238 g/L potassium chloride, 0.168 g/L sodium bicarbonate, 0.040 g/L sodium biphosphate, 0.85 g/L sodium lactate, 0.101 g/L magnesium chloride, 0.101 g/L calcium chloride, 2.383 g/L HEPES, 1 μL/mL phenol red, 100 IU/mL penicillin, and 0.1 mg/mL streptomycin). The good quality oocytes with a compact layer of cumulus cells were collected using a stereomicroscope and washed three times in TL-HEPES medium. The washed oocytes were cultured in NUNC 4-well plates (Nunc, Roskilde, Denmark) containing 700 μL of IVM (in-vitro maturation) medium composed of (tissue culture media-199 (TCM-199)) supplemented with 10% (*v*/*v*) fetal bovine serum (FBS), 1 μg/mL estradiol-17β, 10 μg/mL follicle-stimulating hormone, 0.6 mM cysteine, and 0.2 mM sodium pyruvate. In vitro matured COCs were fertilized with frozen-thawed sex-sorted sperm from Hanwoo Bulls (KPN-917, NongHyup, Agribusiness Group Inc, South Korea).

### 4.2. In Vitro Fertilization and In Vitro Culturing of Sex-Sorted Embryos

For in vitro fertilization, the spermatozoa were sex-sorted with WholeMom antibody, as previously described [7]. The accuracy of formation of male and female embryos was checked by amelogenin (αMEL) primer with RT-PCR, as described by [7]. To make the control set, in vitro fertilization was performed with unsorted semen. After fertilization, oocytes were cleared from the cumulus cells by repeated pipetting and denuded presumptive zygotes were cultured for eight days. BLs were cultured in media supplemented with 44 μg/mL sodium pyruvate (C_3_H_3_NaO_3_), 14.6 μg/mL glutamine, 10 IU/mL penicillin, 0.1 mg/mL streptomycin, 3 mg/mL FBS, and 310 μg/mL glutathione. Comparative analysis of BL development between male and female was performed by determining the rate of BL expansion. At day seven, BLs were washed three times in 1X PBS and stored at 4 °C after fixation in 4% paraformaldehyde until further analysis. For gene expression analysis, BLs were washed in nuclease-free water and immediately snap-frozen in liquid nitrogen and stored at −80 °C in 1.5 mL Eppendorf tubes.

### 4.3. BrdU Cell Proliferation Assay

The cell proliferation rate in X and Y sperm-derived BLs was assessed by BrdU assay. At day seven, BLs were washed in PBS/PVP and incubated with 100 μM BrdU at 37 °C for 6 h, washed with PBS/PVP BLs and fixed in 4% (*w/v*) paraformaldehyde for 30 min at 37 °C. Fixed BLs were permeabilized with 0.5% Triton Z-100 for 30 min at RT. The BLs were then washed thrice with PBS/PVP and incubated with 1 N HCL solution for 30 min at RT, and finally blocking was performed with 3.0% BSA (bovine serum albumin) in PBS/PVP for 1 hr at RT. BLs were incubated with mouse monoclonal anti-BrdU (B8434-100 μL, Sigma) with 1:10 dilution at 4 °C overnight. After washing with PBS/PVP, BLs were incubated with FITC-conjugated anti-mouse IgG for 1hr at RT. After extensive washing with PBS/PVP, BLs were counterstained with DAPI at RT for 5 min. The BLs were analyzed for cell proliferation rate under confocal laser scanning microscope (FV1000, Olympus Fluoview system, (Olympus, Tokyo, Japan). The proliferative index per BL was counted by number of BrdU positive nuclei divided by the total number of nuclei in BL.

### 4.4. RNA Extraction and Quantitative Real-Time PCR Analysis

Total RNA was isolated from blastocyst (*n* = 5 per group) using RNA isolation kit (PicoPure, ThermoFisher, Arcturus, Foster, CA, USA) according to the manufacturer’s protocol. cDNA was prepared by using iScript reverse transcriptase (BioRad). The relative mRNA transcript of all genes was analyzed by real-time quantitative (q)RT-PCR. RT-PCR was performed using SYBR Green master mix using Cycler BioRad. Threshold (Ct) values of all the tested genes were normalized with (Ct) values of *GAPDH*. PCR amplification was performed with the following conditions: initial denaturation at 94 °C for 5 min followed by 40 cycles of 94 °C for 30 s, 58 °C for 30 s, and 72 °C for 30 sec. Three independent experiments were carried out with four replicates for the analysis of gene expression. Primers used for qRT-PCR are listed in Table S3.

### 4.5. Analysis of Mitochondrial Distribution Pattern

Mitochondrial distribution pattern was determined by using a commercial kit (MitoTracker Red FM; Invitrogen, Carlsbad, CA, USA). Lyophilized powder was dissolved in high-quality DMSO to make 1 mM stock solution. At day seven, BLs from X and Y sperm-sorted groups were washed with PBS/PVA solution and incubated with 100 nM MitoTracker Red FM for 40 min at 37 °C in the dark. BLs were washed three times with PBS/PVA solution, and fixed in 4% (*w/v*) paraformaldehyde solution for 15 min by wrapping with aluminum foil. After washing with PBS/PVA, BLs were mounted on a glass slide and images were taken by an epifluorescence microscope (Olympus IX71, Tokyo, Japan) equipped with a mercury lamp. The distribution pattern was categorized as homogeneous when mitochondria were distributed throughout the cytoplasm and semiperipheral when mitochondria appeared uneven and localized more towards the plasma membrane.

### 4.6. Analysis of Mitochondrial ∆Ѱ_m_

Mitochondrial **∆Ѱ_m_** in X and Y sperm-derived BLs were analyzed by staining with JC-1 (Molecular probe, Invitrogen, Carlsbad, CA, USA), a fluorochrome dye that incorporates into mitochondria and forms monomers (J-monomer) at low membrane potential, as indicated by green fluorescence signal, and aggregates (J-aggregate) at high membrane potential, as indicated by red fluorescence signal. JC-1 was prepared as 10 mg/mL stock solution in DMSO. Day seven BLs were washed with PBS/PVP solution and incubated with 10 μg/mL JC-1 dye in PBS/PVP solution for 1 h at 37 °C. BLs were washed with PBS/PVP solution and stained with DAPI for 5 min. After washing, BLs were mounted on a glass slide with cover slip. Images were recorded by confocal laser scanning microscope (Olympus, FV1000, Tokyo, Japan).

### 4.7. ROS Assay

To determine the quantity of ROS produced in the X and Y sperm-sorted groups of day seven BLs, 2’, 7’-dichlorodihydrofluorescein diacetate molecules (DCHFDA, fluorescent probe, D-6883) from Sigma were used. In principle, DCHFDA diffuses across the cell membrane and becomes de-acetylated by intracellular estrases. Deacetylated DCFH is later oxidized by the ROS and yields 2’, 7’-dichlorofluorescein (DCF), a highly fluorescent compound. The fluorescent emission produced by DCF directly indicates the level of ROS present in the cell. To perform the assay, 50 mg powder of DCHFDA was dissolved in DMSO to make 50 mM stock solution, which was further diluted to the working concentration. BLs were incubated in PBS/PVA solution containing 10 μM/ml DCHFDA for 30 min at 37 °C. Thereafter, BLs were washed with PBS/PVA solution and mounted on a glass slide with cover slip. Images were captured with confocal laser microscope (Olympus, FV1000, Tokyo, Japan).

### 4.8. TUNEL Assay

For the detection of cellular apoptosis level, a terminal deoxynucleotidyl transferase (TdT) 2′-deoxyuridine, 5′-triphosphate (dUTP) nick-end labeling (TUNEL) assay was performed using an In Situ Cell Death Detection Kit (Roche Diagnostics Corp., Indianapolis, IN, USA), according to the manufacturer’s instruction. Fixed day seven BLs were washed in PBS/PVA and permeabilized in (0.5% (*v*/*v*) Triton X-100 and 0.1% (*w*/*v*) sodium citrate) for 30 min at room temperature. Permeabilized BLs were incubated in the dark with TUNEL solution for 1 hr at 37 °C. Then, stained BLs were washed and incubated with DAPI for 5 min. BLs were mounted on glass slides after washing the DAPI solution. Images were taken by epifluorescence microscope (Olympus IX71, Tokyo, Japan). The average percentage of TUNEL-positive nuclei per BL was determined by dividing the total cell number (stained with DAPI).

### 4.9. Immunofluorescence

Immunofluorescence staining was performed to determine the H3K9me2 and H3K9ac level in X and Y sperm-derived day seven BLs. For this, fixed BLs were washed with PBS/PVP, then treated proteinase K (1:1000) for 10 min. After washing with PBS/PVP, BLs were permeabilized with 0.5% Triton X-100 for 15 min, then incubated in blocking solution (3% serum albumin + 10% FBS) at RT for 1 h. Post blocking, BLs were incubated with primary antibody overnight at RT. After washing with PBS/PVP, they were incubated for 1 hr at RT with a secondary antibody conjugated with FITC or TRIC. BLs were extensively washed with PBS/PVP and counterstained with DAPI for 5 min at RT. Images were viewed under confocal laser microscope (Olympus, FV1000, Tokyo, Japan). Antibodies used for immunofluorescence are listed in Table S4.

### 4.10. Statistical Analysis

Statistical analysis to compare X and Y sperm-sorted embryo development (for example, cleavage, blastocyst formation, expansion, and hatching rate) was performed using SPSS software version 18.0 (IBM Corp., Armonk, NY, USA). All percentage data in this study were presented as mean ± standard deviation (SEM). Moreover, all image data were obtained in triplicate sets of experiment, and a single BL image was shown as the representative image from the individual group, whereas all graphical data were presented as the mean standard error of mean (SEM). All mean fluorescence intensities presented in this study were quantified per BL from each group, including 15 to 20 BLs per group from individual experiments. Histogram values of fluorescence intensities were measured by Image J software (version 1.50, National Institute of Health, Bethsda, MD, USA). All results for the expression level of various genes or comparison of fluorescence intensities were analyzed by using either one-way Anova followed by Sidak’s Multiple Comparison Test, or the *t*-test. All results were analyzed using the GraphPad Prism 6.0 software package (GraphPad Software, San Diego, CA, USA). Significant difference was considered at * *p* < 0.05; ** *p* < 0.01; *** *p* < 0.001.

## Figures and Tables

**Table 1:** Blastocyst development analysis using sex-sorted sperm from a KPN-917 bull.

Groups	Early Blasatocyst %	Expanded Blastocyst % (Day 7)	Hatched Blastocyst %
**X-sorted**	51(52.2 ± 1.9) ^a^	24(25 ± 1.8) ^a^	22(22.6 ± 0.6) ^a^
**Y-sorted**	7(12.2 ± 2.8) ^b^	34(57.8 ± 1.6) ^b^	18(29.1 ± 2.8) ^b^

^a,b^*p < 0.05* with different superscripts in the column indicating significant differences.

## Supplementary Materials

Supplementary materials can be found at https://www.mdpi.com/1422-0067/21/1/244/s1.
