# Supplementary material for: Difference in Developmental Kinetics of Y-Specific Monoclonal Antibody Sorted Male and Female In Vitro Produced Bovine Embryos
Source: Int J Mol Sci. 2019 Dec 30;21(1):244. doi: 10.3390/ijms21010244 (PMC6981608; doi:10.3390/ijms21010244)
Supplement: Supplementary file 1 [file ijms-21-00244-s001.pdf]

Difference in developmental kinetics of Y-specific monoclonal antibody sorted male and female in-vitro produced bovine embryos

SUPPLEMENTARY MATERIALS:

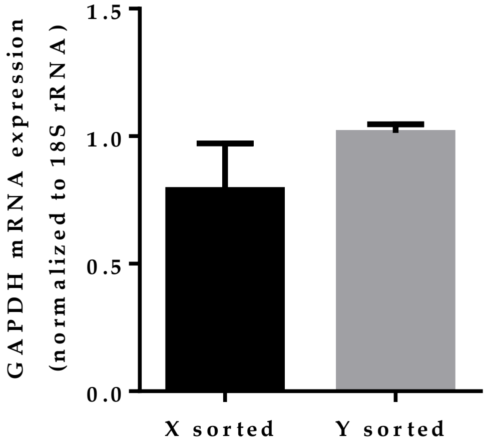

FigureS1: Analysis of *GAPDH* expression stability in X and Y sorted embryos. GAPDH expression in both X and Y sorted embryos were normalized with 18S RNA gene.

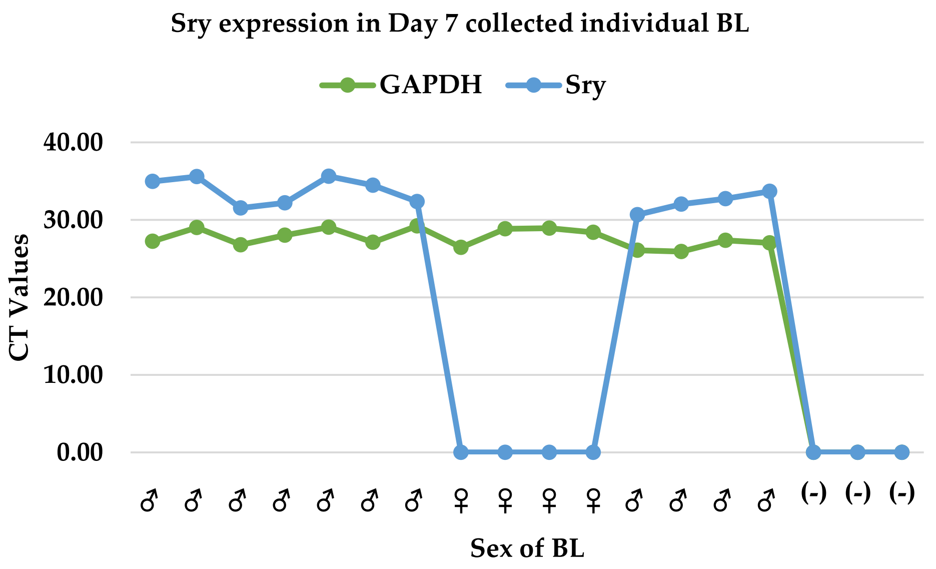

(a)

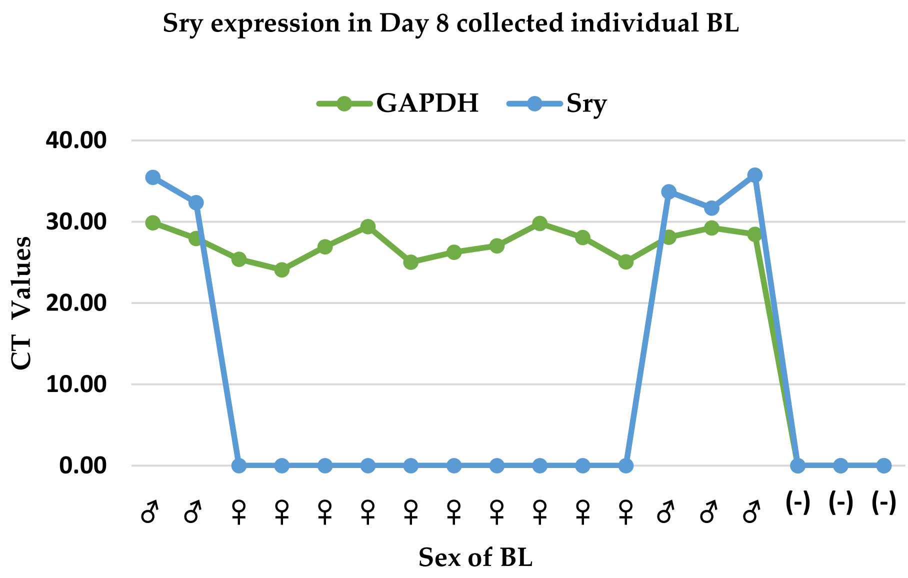

(b)

Figure S2. qRT-PCR analysis for determining the ratio of Y BL from Day 7and 8 expanded BLs. (a-b) Representative graph showing the expression of *Sry* male specific gene from extracted genomic DNA of single BL. Occurrence of males and females in day 7 and 8 collected BLs is represented by Symbols (♂-male) and (♀-female). (-) indicates negative control.

Table S1: Percentage of Cleavage and Blastocyst produced from unsorted and sex sorted sperm.

Groups	Oocytes, n	Speculated Zygote, n	Cleaved Embryos, n %	Total Blastocyst %
**Unsorted**	285	279	218(78 ± 0.3) [a]	78(28 ± 0.4) [a]
**X sorted**	441	423	221(52.3 ± 1.1) [b]	97(23.1 ± 0.8) [b]
**Y sorted**	413	386	180(46.7 ± 0.6) [c]	59(15.7 ± 1.0) [c]

[a,b] $p<0.05$ with different superscript in the column indicate significant difference.

Table S2: Percentage of male and female BL developed at day 7 and day 8 of post-fertilization.

Oocytes, (n)	Total BL obtained	Analyzed Expanded BL		% of ♂	% of ♀
689	205(29%)	Day 7	65	47(72%)	18(27%)
		Day 8	60	19(31%)	41(68%)

Table S3: List of qRT-PCR primers.

Gene Name	Forward	Reverse	Accession #
GAPDH	TTCAACGGCACAGTCAAGG	ACATACTCAGCACCAGCATCAC	NM_001034034
Oct4	AGGTGTTCAGCCAAACGACTAT	GTCTCTGCCTTGCATATCTCCT	NM_174580
IGF-1R	GATCCCGTGTTCTTCTACGTTC	CAGCCTGCTGCTATTTCTTTTT	NM_001244612.1
Ndufa9	ATGGGGTCACAGGTGATTGT	TCCCGTTCCAGTCCATAAAG	NM_205817
Nduv1	GCCATGACTGGAGGCTTAAA	ATTTAAGGCCAGTGGGGAAG	NM_174808.1
Cox4i	CATGTTCTTCATCGGCTTCA	TCATGTCGAGCATCCTCTTG	BT021029
Cox5b	TCACCAACAAGCGGATAGTG	TGTGGCACCAGCTTGTAATG	NM_001034046.2
Glut1	ATCCTCATTGCCGTGGTGCT	ACGATGCCAGAGCCGATGGT	M60448
Glut3	CGCCTTTGGCACTCTCAAC	GCACTGGATGATGGCTGGTAA	AF308829
Glut4	CCACCAGGCACACTTACCACA	CTCTTCCTTCCCAGCCACTGA	U18105
G6PD	ACACCAAGATGATGACCA	GAGCTTCACGTTCTTGTATCG	NM_001244135.1
HPRT	GAAGAGCTACTGTAACGACCA	AAAGTCTGCATTGTCTTCCC	AF176419.1
PGK	CCTGTGGGTGTATTTGAATGG	AGCACTTTACCTTCCAGGAG	BT021601.1
XIST	CTGCTAATCGTTTGTGTTGTG	ATACCATTTCCTTTGCCTGTC	AF104906.5
DNMT1	GTAAGATAGTGGTTGAGTTC	AGAATCCTCTGTGAATCG	AY173048.1
DNMT3a	ATCAAAGAAGACCCCTGGAACT	AATTCCTGGTCATGGTTGTTGG	AY271299
DNMT3b	AAGCAACCAGAGAATAAGACGC	AAGCCATTTGTTCTCGACTCTG	NM_181813
18SrRNA	5'-AGAAACGGCTACCACATCCA-3'	5'-CACCAGACTTGCCCTCCA-3'	AF176811
Sry	GCCAAGACCACATACTCACAAA	AATCCGTGTAGCCAATGTTACC	AB039748.1

Table S4: List of Antibodies used.

Target	Cat #	Manufacturer
Caspase-3	Sc-1225	Santa Cruz
H3K9me2	Ab-1220	Abcam
H3K9ac	Sc-518011	Santa Cruz
DAPI	62248	Thermo Fisher scientific
FITC	B2119	Santa Cruz
TRITC	A6071	Invitrogen
